# Endoscopic Removal of Granular Cell Tumors of Stomach: Case Report and Review of Literature

**DOI:** 10.4021/gr588w

**Published:** 2014-01-15

**Authors:** Arunkumar Krishnan, Ravi Ramakrishnan, Maya Menon

**Affiliations:** a Department of Gastroenterology, Apollo Hospitals, Chennai, India; b Department of Pathology, Apollo Hospitals, Chennai, India

**Keywords:** Granular cell tumors, Stomach, Endoscopic ultrasound, Submuscosal resection

## Abstract

Gastrointestinal granular cell tumors (GCTs), usually benign, soft-tissue tumors, are thought to arise from Schwann cells that may occur at many sites. Only 5-7% of these lesions are detected in the gastrointestinal tract. Histologically, it is composed of sheets or nests of plump round or polygonal cells having abundant slightly amphophilic granular cytoplasm with centrally located uniform pyknotic nuclei and immunohistochemical staining for S-100 protein supports the proposed derivation from Schwann cells. In this study, we reported a case of a solitary GCT of the stomach that was completely removed after endoscopic submuscosal resection.

## Introduction

Granulosa cell tumors (GCTs) are uncommon, usually benign, soft-tissue tumors rarely seen in clinical practice. In the past, they were called granular cell myoblastoma because of suspected muscle origin. Almost always benign, GCTs are found in patients of all ages with equal frequency in both sexes and it appears to be a relatively greater prevalence in blacks than in whites [1]. Although almost any organ may be involved, 70-80% of GCTs appear as small asymptomatic masses in the skin, subcutaneous tissue, or mouth, particularly on the tongue [1]. The onset of this tumor in the gastrointestinal (GI) tract is rare. Almost, 8% of all GCTs occur in the GI tract and the most common location is the esophagus and large intestine [2]. We report a case of a woman with a solitary GCT of the stomach, incidentally found ahead GI endoscopy, which was completely removed endoscopically.

## Case Report

**Figure 1:**
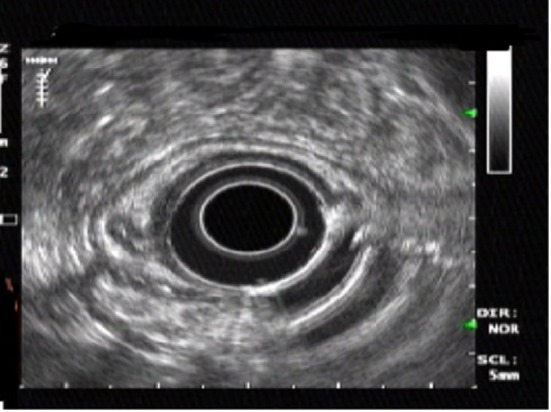
Endoscopic ultrasonography showing a homogeneous, hypoechoic, clearly demarcated mass in the submuscosal layer.

A 54-year-old Nigerian woman was referred to our department for further evaluation of abdominal pain. She had no remarkable past medical history and no history of alcohol consumption, smoking and drugs. She underwent endoscopic examination during a routine checkup. Upper GI endoscopy was performed and revealed a small submucosal lesion of about 1.2 × 1 cm in diameter, located on the lesser curvature of the gastric antrum. The esophagus, duodenum and the remaining parts of the stomach were normal. Upon hospitalization, physical examination, biochemical parameters were completely normal. Endosonography demonstrated a homogeneous, hypoechoic, clearly demarcated 1.2 × 1 cm mass, which was confined to the submucosal layer and above the muscularis propria (Fig. 1). It was challenging to confirm the diagnosis. Because the tumor was relatively small in size, it was considered for endoscopic resection.

**Figure 2:**
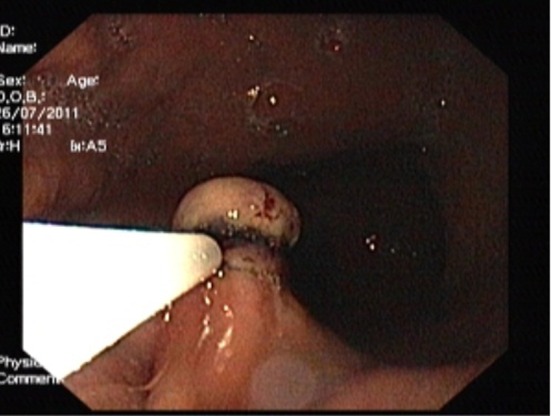
Endoscopic image showing the tumor was lifted with placement of elastic bands over the lower end of the tumor.

Hypertonic saline-epinephrine solution was injected to distinct the tumor from the muscularis propria layer and prevent bleeding. The tumor was lifted with placement of elastic bands over tissue to produce mechanical compression over the lower end of the tumor (Fig. 2) and cut electrically with a high-frequency snare inserted, and submucosal resection was done. No post procedural complication, such as bleeding or perforation. The excised specimen showed complete removal of the lesion. Cross-sections showed the tumor to be well-defined, homogeneous, solid and of yellowish color.

**Figure 3:**
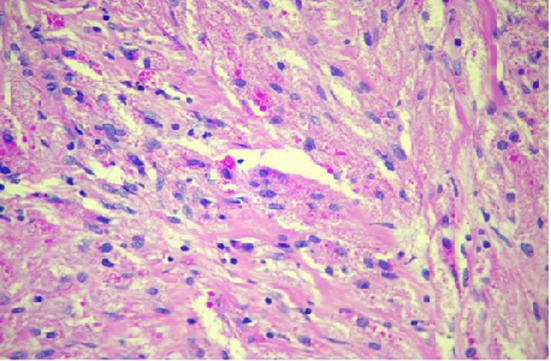
Granular cell tumor hematoxylin and eosin stain showing sheets of polygonal cells with coarsely granular eosinophilic cytoplasm and small vesicular nuclei.

**Figure 4:**
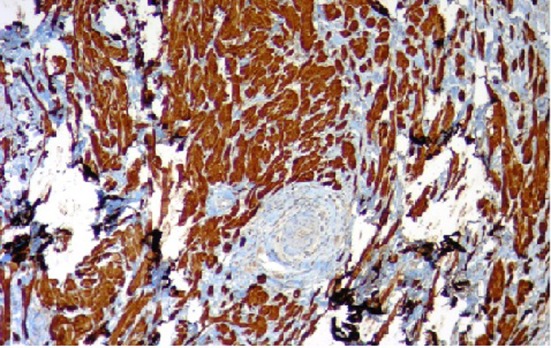
Immunohistochemical staining for S-100 protein.

In the resected specimen, the tumor measured 1.2 × 0.6 × 0.5 cm in diameter. Histologic appearance showed submucosa to contain a lesion which is circumscribed and composed of nets and fascicles of cells with abundant granular cytoplasm and vesicular nuclei. Wisp of collagen is seen intersecting the lesion (Fig. 3). The granules were positive for periodic acid-Schiff stain, and also were immunoreactive to NSE and S-100 (Fig. 4). The diagnosis of GCT was made. The post procedure recovery was uneventful. She remained asymptomatic and no recurrent disease was observed after a 1-year follow-up.

## Discussion

GCTs were defined for the first time by Abrikosoff in 1926. GCTs occur as intramural lesions throughout the GI tract.

It has become obvious that they may occur at many sites, although they affect most frequent skin or subcutaneous tissues of the chest and upper extremities, tongue, breast, female genital organs and only rarely the GI tract [3]. At least, half of the patients were black.

In addition, in half of cases, gastric GCT proved to be associated with esophageal synchronous localized and was rarely associated with other benign or malignant gastric diseases. Similar to our case, only three cases were reported in the world literature; gastric GCT did not illustrate multiple locations or was related to other gastric lesions [4].

The tumor presents as a small nodule or plaque with grayish-white to yellow color endoscopically and usually not greater than 2 cm that originate from the deep mucosa or submucosa [3, 5]. On cut section, GCTs are pale, yellow-tan or yellow-gray. The cells are of Schwann cell origin, rounded, polygonal or spindled, and have a small/rounded nucleus [6].

Histologically, GCT is composed of sheets or nests of plump round or polygonal cells having abundant slightly amphophilic granular cytoplasm with small, round, centrally located uniform pyknotic nuclei [7]. Immunohistochemical staining for S-100 protein supports the proposed origin of the tumor from Schwann cells and myelin proteins [7, 8]. GCTs show immunoreactivity also for vimentin, NSE, CD68 and CD57 [9, 10]. Recently, Parfitt et al [11] demonstrated expression of an intermediate filament protein called nestin (found normally in neuroectodermal stem cells and early skeletal muscle) in GCTs, some of which were located in the esophagus [12]. Nestin might be regarded as a useful marker for identifying GCTs.

There is controversy concerning the histogenesis of GCTs, thus several synonyms have been used to describe this entity. Myoblasts, Schwann cells, histiocytes, perineural fibroblasts and undifferentiated mesenchymal cells have been postulated as the origin of the tumor [13], while theories of the non-neoplastic nature of the lesion that result from trauma, as a degenerative process, or as a storage disorder involving histiocytes have also been considered. However, recent studies support a peripheral nerve-related cell of origin for the majority of these tumors based on the findings of cytoplasmic granules with numerous membrane-bound vacuoles containing myelin-like tubules and “angulate bodies” that show a close relation with pre-existent axons at the ultrastructural level, found between granular cells [13]. The expression of nestin in GCTs suggests that these tumors may arise from a common multipotential stem cell in the GI tract, which has the capability to differentiate along both interstitial cells of Cajal and peripheral nerve pathways [14].

GCTs are generally benign neoplasms, and malignancy rate is estimated to be 1 to 3% of all lesions [13]. There are reports of cases that have recurred or metastasized despite having a benign histologic appearance [13]. Individualities of malignant GCTs are local recurrence, large size (> 4 cm), rapid growth, invasion of adjacent organs and involvement of multiple layers in the GI tract [3, 14]. Histologic features of malignant GCTs include necrosis, spindling, vesicular nuclei with large nucleoli, high nucleocytoplasmic ratio, cellular pleomorphism and increased mitotic activity [15, 16].

Endoscopic ultrasonography has recently been used more frequently for determining the depth of tumor invasion in the GI wall, and may also be useful to evaluate GI tract submucosal tumors [2]. On EUS, GCTs usually arise in the lamina propria or deep mucosa layers of the GI tract, are usually less than 3 cm, hypoechoic, mildly inhomogeneous, and have smooth margins if benign. They are usually slightly more echogenic than leiomyomas [17, 18]. Tada et al [14] stressed that the treatment of choice for GCT should be determined by EUS findings; the tumor is amenable to endoscopic treatment when EUS shows that the tumor is localized in the submucosa and has not invaded the muscularis propria. If the tumor is initially separate from the muscularis propria, the distance between tumor and muscularis propria can be increased by injecting the solution and lifting the lesion, after which removal can be carried out more safely and completely.

Yasuda et al [19] used techniques to increase the distance of the the tumor by injecting saline if it is attached to muscularis propria. However, saline injection alone with band ligation in the lower end of the tumor can be useful for complete removal of tumor from the base. The tumor is drawn into the banding device with suction, and then the rubber band is placed around the tumor. Ligation takes place when the suction is applied over the tumor. The tissue should be appropriate for suction. In fibrotic and hard mucosal tissue, although suction is successful, later the band may release from the site.

In summary, EUS is very helpful in evaluating GCTs to achieve a tissue diagnosis and to evaluate for possible resection of the tumor. Saline injection along with band ligation in the base of the tumor helps to do the complete removal of tumor without complications. Major surgical resection is probably unnecessary when a small submucosal tumor is detected in the stomach of a patient. When the excised tissue reveals findings of malignancy, further surgical intervention ought to be considered.

## References

[1] Johnston J, Helwig EB (1981). Granular cell tumors of the gastrointestinal tract and perianal region: a study of 74 cases. Dig Dis Sci.

[2] Nakachi A, Miyazato H, Oshiro T, Shimoji H, Shiraishi M, Muto Y (2000). Granular cell tumor of the rectum: a case report and review of the literature. J Gastroenterol.

[3] Eisen RN, Kirby WM, O'Quinn JL (1991). Granular cell tumor of the biliary tree. A report of two cases and a review of the literature. Am J Surg Pathol.

[4] Patti R, Almasio PL, Di Vita G (2006). Granular cell tumor of stomach: a case report and review of literature. World J Gastroenterol.

[5] Yasuda I, Tomita E, Nagura K, Nishigaki Y, Yamada O, Kachi H (1995). Endoscopic removal of granular cell tumors. Gastrointest Endosc.

[6] Joshi A, Chandrasoma P, Kiyabu M (1992). Multiple granular cell tumors of the gastrointestinal tract with subsequent development of esophageal squamous carcinoma. Dig Dis Sci.

[7] Weiss S, Goldblum JR (2001). Enzinger and Weiss's Soft Tissue Tumors.

[8] Wiech T, Walch A, Werner M (2005). Histopathological classification of nonneoplastic and neoplastic gastrointestinal submucosal lesions. Endoscopy.

[9] Mazur MT, Shultz JJ, Myers JL (1990). Granular cell tumor. Immunohistochemical analysis of 21 benign tumors and one malignant tumor. Arch Pathol Lab Med.

[10] Kurtin PJ, Bonin DM (1994). Immunohistochemical demonstration of the lysosome-associated glycoprotein CD68 (KP-1) in granular cell tumors and schwannomas. Hum Pathol.

[11] Parfitt JR, McLean CA, Joseph MG, Streutker CJ, Al-Haddad S, Driman DK (2006). Granular cell tumours of the gastrointestinal tract: expression of nestin and clinicopathological evaluation of 11 patients. Histopathology.

[12] Sarlomo-Rikala M, Tsujimura T, Lendahl U, Miettinen M (2002). Patterns of nestin and other intermediate filament expression distinguish between gastrointestinal stromal tumors, leiomyomas and schwannomas. APMIS.

[13] Ordonez NG, Mackay B (1999). Granular cell tumor: a review of the pathology and histogenesis. Ultrastruct Pathol.

[14] Tada S, Iida M, Yao T, Miyagahara T, Hasuda S, Fujishima M (1990). Granular cell tumor of the esophagus: endoscopic ultrasonographic demonstration and endoscopic removal. Am J Gastroenterol.

[15] Jardines L, Cheung L, LiVolsi V, Hendrickson S, Brooks JJ (1994). Malignant granular cell tumors: report of a case and review of the literature. Surgery.

[16] Yoshizawa A, Ota H, Sakaguchi N, Kanai S, Nakayama J, Matsuzawa K, Tsuzuki S (2004). Malignant granular cell tumor of the esophagus. Virchows Arch.

[17] Palazzo L, Landi B, Cellier C, Roseau G, Chaussade S, Couturier D, Barbier J (1997). Endosonographic features of esophageal granular cell tumors. Endoscopy.

[18] Polkowski M (2005). Endoscopic ultrasound and endoscopic ultrasound-guided fine-needle biopsy for the diagnosis of malignant submucosal tumors. Endoscopy.

[19] Yasuda I, Nakazawa S, Yoshino J (1993). Indication for endoscopic resection of submucosal tumor by endoscopic ultrasonography. Gastroenterol Endosc (Jpn).

